# Anorexia

**DOI:** 10.1093/emph/eou026

**Published:** 2014-11-05

**Authors:** Joe Alcock, Edmund K. LeGrand

**Affiliations:** ^1^Department of Emergency Medicine, University of New Mexico, Albuquerque, NM, USA; ^2^Department of Biomedical and Diagnostic Sciences, College of Veterinary Medicine, University of Tennessee, Knoxville, TN, USA

## Anorexia of illness

During infection it is very common to lose one's appetite (anorexia) and reduce nutrient intake. Anorexia is one of a group of symptoms collectively termed sickness behaviors, which in turn are a part of the acute-phase response (APR). The APR and its components, including anorexia, fever and iron sequestration, are induced by pro-inflammatory cytokines, e.g. interleukin-6, interleukin-1 and tumor necrosis factor-α [1, 2]. Whether anorexia in infection is adaptive, or merely a secondary side effect of inflammation, is controversial since one would expect *increased* food intake is required to meet the nutritional needs of a strong immune response.

Uncertainty exists whether to give more or less nutritional support for critically ill patients [3]. However, two recent randomized controlled trials showed improved survival [4] and fewer complications [5] with lower calorie delivery. Animal studies have shown similar results: *Listeria*-infected mice had higher mortality when force-fed to pre-infection nutrition levels [6]. 

## Evolutionary perspectives

Hart [7] proposed that anorexia acts in concert with iron sequestration in the APR, depriving pathogens of growth-limiting micronutrients. Straub *et al.* [8] argued instead that the main benefit of anorexia during inflammation is the redistribution of energy to the activated immune system, away from abdominal organs, muscles and the brain. Both Hart and Straub posited that energy conservation is an important consequence of anorexia, and of sickness behavior generally. By avoiding the costs of foraging and digestion, energy is freed up for immune defense [7, 8].

The notion that anorexia conserves energy seems problematic because eating typically increases net energy. Although the nutritional stress of anorexia harms the host, we have proposed that it causes even more harm to rapidly dividing pathogens and infected host cells weakened by pathogens [9]. In the immune brinksmanship model, anorexia is conceptualized as a gamble that the host can withstand nutrient and energy deprivation better than the invading organisms [9]. Differences in fitness between pathogens and healthy host cells, amplified by anorexia and other APR stressors, could explain the evolution of anorexia [9]. Recent mathematical modeling indicates that anorexia can be beneficial, particularly when pathogens have prioritized access to dietary energy [10].

## Future implications

The idea of ‘underfeeding’ critically ill patients is controversial and awaits a definitive trial. Managing illness anorexia should include a careful cost-benefit analysis that considers the baseline nutritional status, infection risk and the costs of malnutrition. Until evolutionary tradeoffs involving anorexia are understood, there will not be a rational basis for optimizing patient nutrition during illness.
